# Solid State NMR a Powerful Technique for Investigating Sustainable/Renewable Cellulose-Based Materials

**DOI:** 10.3390/polym14051049

**Published:** 2022-03-06

**Authors:** Mustapha El Hariri El Nokab, Mohamed H. Habib, Yasser A. Alassmy, Marwan M. Abduljawad, Khalid M. Alshamrani, Khaled O. Sebakhy

**Affiliations:** 1 Zernike Institute for Advanced Materials (ZIAM), University of Groningen, 4, 9747 AG Nijenborgh, The Netherlands; m.el.hariri.el.nokab@rug.nl; 2 Department of Microbiology and Immunology, Faculty of Pharmacy, Cairo University, Kasr El-Aini St., 11562 Cairo, Egypt; mohamed.habib@pharma.cu.edu.eg; 3 King Abdulaziz City for Science and Technology (KACST), Riyadh 11442, Saudi Arabia; yalassmy@kacst.edu.sa (Y.A.A.); mabduljawad@kacst.edu.sa (M.M.A.); kshamrani@kacst.edu.sa (K.M.A.); 4 Engineering and Technology Institute Groningen (ENTEG), University of Groningen, 4, 9747 AG Nijenborgh, The Netherlands

**Keywords:** solid state NMR spectroscopy, 1D ^13^C CP MAS, 2D ^13^C correlation, cellulose-based materials, sustainability, solvent–matrix interactions

## Abstract

Solid state nuclear magnetic resonance (ssNMR) is a powerful and attractive characterization method for obtaining insights into the chemical structure and dynamics of a wide range of materials. Current interest in cellulose-based materials, as sustainable and renewable natural polymer products, requires deep investigation and analysis of the chemical structure, molecular packing, end chain motion, functional modification, and solvent–matrix interactions, which strongly dictate the final product properties and tailor their end applications. In comparison to other spectroscopic techniques, on an atomic level, ssNMR is considered more advanced, especially in the structural analysis of cellulose-based materials; however, due to a dearth in the availability of a broad range of pulse sequences, and time consuming experiments, its capabilities are underestimated. This critical review article presents the comprehensive and up-to-date work done using ssNMR, including the most advanced NMR strategies used to overcome and resolve the structural difficulties present in different types of cellulose-based materials.

## 1. Introduction

Current environmental constraints, along with the depletion of fossil fuels, necessitate the usage of greener materials in society [1,2]. Cellulose is among the most renewable, abundant, and ubiquitous biopolymer/biomass available in nature [1,2,3,4,5,6,7,8,9,10,11], as it constitutes one of the major components of the cell walls in green plants and is responsible for mechanical strength enhancement [3,4,5]. This polysaccharide was discovered by the French chemist Anselme Payen in 1838 via isolation from plant matter and has the formula (C_6_H_10_O_5_)_n_, where n represents the degree of polymerization [12]. It consists of linear polymer chains that are composed of several hundred to many thousands of β(1→4) linked D-glucose units [13].

Cellulose structure is peculiar, in that it has a d-glucose unit at one end, a C4-OH group as the reducing end, and a C1-OH as terminating group. Some celluloses contain extra carbonyl and carboxyl groups, such as bleached wood pulp [4]. The cellulose present in plants is usually found in and amongst a mixture of hemicellulose, lignin, pectin, and other substances. The unique molecular structure of cellulose gives it interesting properties, such as chirality, hydrophilicity, degradability, and high reactivity, due to the presence of the donor, OH group [7,8,10]. As a result of the strong hydrogen bonds present in cellulose, those materials exhibit crystalline fiber structures [4]. More interestingly, and contrary to other polysaccharides, cellulose can retain a semi-crystalline state of aggregation in aqueous environments [14,15]. From a microstructure perspective, cellulose consists of fibrils with crystalline and amorphous domains [4]. Cellulose can be classified and segmented as follows,

- bacterial cellulose [16], preparation method: Gluconacetobater hansenii bacteria using corn steep liquor as nutrient,
- cellulose ester (e.g., cellulose acetate) [17], preparation method: reaction of cellulose with acetic anhydride and acetic acid in the presence of sulfuric acid,
- ethyl cellulose (EC) [18,19,20], preparation method: etherification of cellulose obtained from wood pulp or cotton in an alkaline medium,
- hydroxypropyl cellulose (HPC) [21,22,23], preparation method: reaction of alkali cellulose with propylene oxide at elevated pressure and temperature.

Although bacterial cellulose is the purest form of cellulose and can be isotopically labelled to ease its characterization, its production cost is high, requires expensive culture media, and is obtained in low yields [16,24]. Cellulose acetate is an ester of cellulose and finds various applications in films, membranes, and fibers, depending on the way it is processed [17]. Ethyl cellulose [25,26] has also found applications in drug delivery systems [19]. Furthermore, hydroxypropyl cellulose, which is soluble in water, has been used as a lubricant [27,28].

Cellulose and its derivatives have numerous applications, but cellulose itself is most commonly used in the paper and pulp industry. It is also a major component present in natural fibers [29]. Important applications of cellulose include the production of biofuel [30], viscous fibers [31], and use as a thickening agent in the food industry [32]. Alternative applications of cellulose include, but are not limited to, tissue engineering [15,33], drug delivery [34,35], nanoadsorbents for proteins [9], wastewater treatment [36], sensor technology [10], and biomedical applications [37].

Among the techniques that have been widely used to determine the degree of crystallinity of cellulose-based materials is X-ray diffraction (XRD) [38,39,40]. This technique was applied to cellulose crystallites present in flax textile fibers [38]. Wide angle X-ray diffraction (WAXD) was utilized to measure the crystallite thickness before and after bleaching [38]. The overall crystallinity decreased after treatment, as was evidenced by WAXD [38]. However, when using ^13^C-ssNMR as a characterization technique the cellulose crystallinity slightly increased [38]. This was attributed to the difference in chemical selectivity between both techniques. Low spin–spin relaxation times and an increase in crystallite thickness after bleaching confirmed the strong adhesion between cellulose crystallites, hemicelluloses, and pectins [38]. Moreover, in another study, wide angle X-ray scattering (WAXS) and ^13^C-ssNMR were used to determine the diameter of microfibrils of celery collenchyma cellulose [40]. However in that work, both WAXS and ^13^C-ssNMR used an uncertain assumption that the surface chains were positioned within an extension of the crystalline lattice. Another study combined attenuated total reflection Fourier transform infrared spectroscopy (ATR-FTIR), XRD, transmission electron microscopy (TEM), and ^13^C-ssNMR for four different types of cellulose microfibrils that were obtained from vascular bundles of banana rachis and treated with different potassium hydroxide (KOH) concentrations [41].

Over the last decades, significant effort has been made to revolutionize solid state NMR, from a low resolution out of sight technique into an indispensable one for structural and dynamic determination of a wide range of materials in different physical states [42]. NMR spectra for solids are known for their broadness, broad line width and weak intensity, which is typically attributed to the interactions between the active nuclear spins and the magnetic fields [43]. The emerging orientation-dependent nuclear magnetic interactions in solid states are formed due to the restricted thermal motions and lack of rapid molecular tumbling. This poor dynamical motion unveils different types of internuclear and orientation-dependent nuclear interactions, which hold detailed information on the local geometric and electronic structure [44,45,46]. Different solid-state NMR techniques have been developed, including newly designed pulse sequences, to suppress and eliminate the broadness in the spectra of solid materials [47]. Moreover, the combination of solid state NMR and magic angle spinning (MAS) technique was the most effective in suppressing the anisotropic and dipolar interactions dominant in solid state. This is where a sample is subjected to a rapid rotation along an axis aligned at an angle of 54.47° with respect to the static external magnetic field [48].

**Scheme 1:**
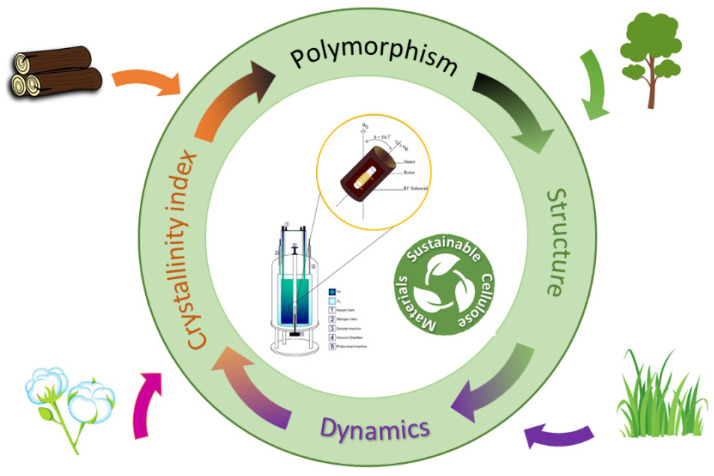
ssNMR, an advanced technique for the characterization of sustainable cellulose-based materials.

Currently, the incorporation of the MAS technique in ssNMR is capable of improving the resolution and spectral signal-to-noise ratio; thus, providing essential chemical information at an ultrastructural level for cellulose-based materials in various environments, as depicted in Scheme 1 [49]. Nevertheless, for further signal enhancement, especially for low sensitivity nuclei, e.g., ^13^C, cross polarization (CP) is required [50]. CP is not only used to extract structural data for polymeric materials, but can also supply unique knowledge on the molecular interactions, polymorphism, and dynamics [51,52,53,54]. 1D ^13^C CP MAS experiments benefited from the combination of different solid state NMR techniques to predominate over all other techniques. Examples of such techniques are: (a) cross polarization, where magnetization is transferred from a high natural abundance and gyromagnetic ratio nuclei, e.g., ^1^H into the low natural abundance and gyromagnetic ratio nuclei e.g., ^13^C; thus, enhancing the intensity of the ^13^C spectra; (b) magic angle spinning, where anisotropic interactions are averaged and the spectra tend to gain resolution; and (c) high power ^1^H decoupling, which reduces the line broadening by disconnecting ^1^H spin systems from ^13^C ones [52,55].

The modern solid state NMR techniques used to study cellulose, lignocellulose biomass, plant cell walls, and other cellulose derivatives include 1D ^13^C CP MAS and 2D ^13^C correlation experiments. Table 1 summarizes the modern solid state NMR pulse sequences used in cellulose-based research and applications:

## 2. Cellulose Structure Investigated via Solid State NMR Spectroscopy

After decades of research on cellulose-based nature materials using different diffraction and spectroscopy techniques, the exact structure of cellulose in natural products is still not fully understood. One of the most useful approaches for the analysis of the cellulose structure on an atomic level is using solid state NMR spectroscopy with 1D ^13^C CP MAS and 2D ^13^C INADEQUATE (Incredible Natural Abundance Double Quantum Transfer Experiment) techniques, due to their sensitivity to chemically equivalent nuclei in magnetically non-equivalent environments (crystalline and amorphous cellulose showing different chemical shifts).

### 2.1. Pure Cellulose with ^13^C CP MAS and Different Crystal Phases

**Figure 1:**
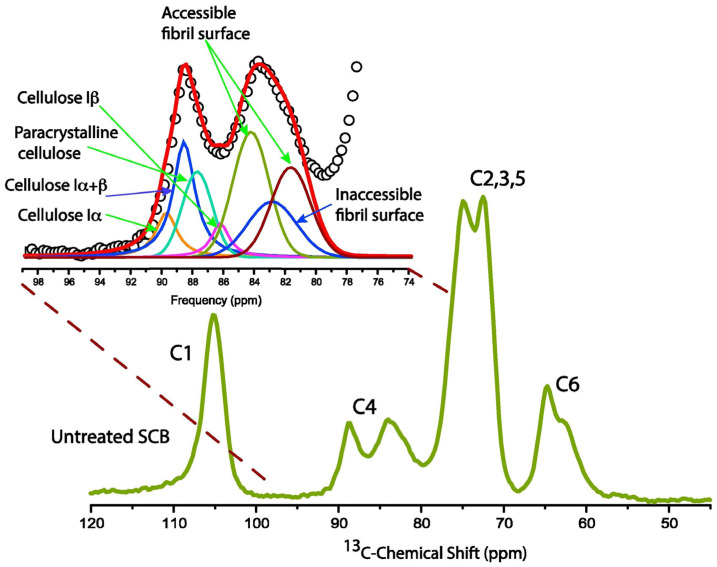
1D ^13^C CP MAS NMR spectrum of untreated sugarcane bagasse (SCB). C_4_ region shows the most significant spectral changes. Inset on top of the spectrum shows the spectral fitting and deconvoluted peaks of C_4_ region. Reproduced with permission. Ref. [72] Copyright 2019, Elsevier.

Different cellulose materials obtained from pulp (cellulose I) [86,87,88], regenerated cellulose fibers (cellulose II) [65], cellulose-based shells [89], and bacterial cellulose [59,90] were characterized by ^13^C CP/MAS solid state NMR [91]. The ^13^C CP MAS NMR spectrum was extended over a chemical shift from 57 to 108 ppm and divided into four separate regions, resolved to a different extent, including less resolved regions C_6_ (57–67 ppm), C_1_ (102–108 ppm) and C2,3,5 in the cellulosic ring (70–80 ppm), and well resolved region C_4_ (80–92 ppm) [72]. Ultra-structural information regarding crystallinity could be extracted from the C4 peak in the isolated cellulose spectrum, which is resolved into one crystalline and another amorphous region. When spectral deconvolution was applied to the C4 region of untreated sugarcane bagasse, several signals were recorded (Figure 1) that were a sum of four signals and were assigned to the crystalline region possessing narrow fitting and sharpness, corresponding to α (90 ppm), β (86 ppm), a mixture of α + β cellulose (89 ppm) crystal structures and paracrystalline cellulose (88 ppm). To the right, the signal for inaccessible fibril surface appears at (83 ppm) and two other signals (84 and 82 ppm) are assigned to accessible fibril surface [72]. The cellulose amorphous region consists of two characteristic doublets at 84 and 85 ppm, corresponding to the accessible fibril surface and identified as located on top of the two different crystal planes, with a broad signal for deeply located inaccessible fibril surface at round 85.4 ppm [92]. The spatial distribution of the cellulose morphologies within the cellulose microfibril suggests a core-shell structure, consisting of an outer amorphous shell composed mainly of accessible cellulose microfibril surfaces [49].

### 2.2. Cellulose Polymorphism in Plant Primary Cell Walls

**Figure 2:**
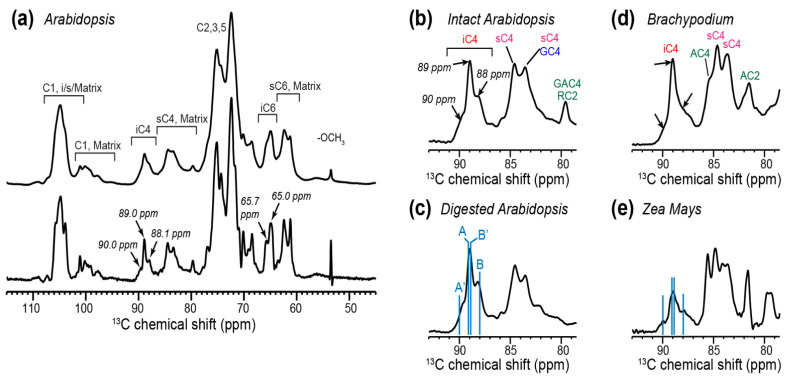
1D ^13^C CP MAS NMR spectra of primary cell walls with high magnetic fields. (**a**) Undried Arabidopsis cell walls without (top) and with (bottom) resolution-enhanced window function. (**b**) Intact Arabidopsis cell wall. (**c**) Partially digested Arabidopsis cell wall. (**d**) Brachypodium cell wall. (**e**) Zea mays cell wall. Only the C4 region of the ^13^C spectra of various cell walls is shown in (**b**–**e**). Blue lines in (**c**,**e**) indicate the crystalline cellulose C4 chemical shifts in Iα (A and A′) and Iβ (B and B′) allomorphs. Reproduced with permission. Ref. [69] Copyright 2016, The American Chemical Society.

Wang et al. [69] managed to explain cellulose polymorphism in plant primary cell walls in different plants, including Arabidopsis, Brachypodium, and Zea Mays. Using the data generated by the ^13^C NMR spectra of these plants, a better picture of such polymorphism was shown. Figure 2a–c show the 1D ^13^C NMR spectra of Arabidopsis in different conditions, whereas Figure 2d,e show the ^13^C NMR spectra of Brachypodium and Zea Mays, respectively. Figure 2a illustrates the 1D ^13^C CP MAS spectrum of Arabidopsis primary cell walls. Despite the usage of ultra-high magnetic fields, overlapping still dominates the main shifts at 105 ppm (C1) and at 70–75 ppm (C2,3,5). Meanwhile, applying a resolution-enhanced window function significantly resolves the interior carbon peaks (iC4 and iC6) found in the polysaccharide signals, and several structures can be seen. Such a resolution-enhanced window function reveals that the iC4 peak at 89 ppm consists, not one, but three partially resolved peaks, and the iC6 peak at 65 ppm consists of two peaks. A detailed look at the iC4 shows the superposition of the Iα allomorph’s C4 chemical shifts (90 and 89 ppm) and the Iβ C4 chemical shifts (89 and 88 ppm), as shown in Figure 2b,c. The presence of such features in Arabidopsis, Brachypodium, and Zea Mays suggests that structural polymorphism is not uncommon in plant primary-wall cellulose. Digestion of Arabidopsis (Figure 2c) reveals the fact that the fine chemical structural features represented in the iC4 shifts were not affected, indicating that the primary cellulose structure is robust [69].

**Figure 3:**
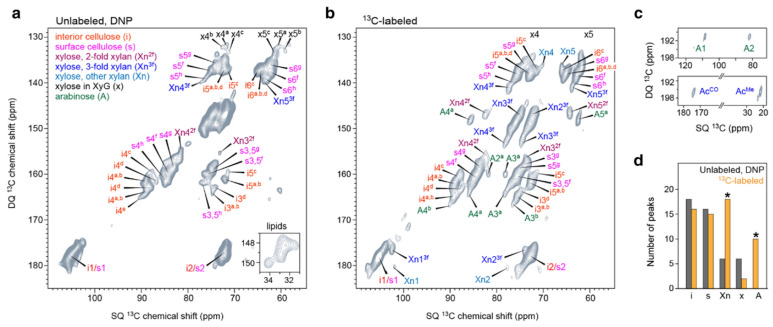
2D ^13^C–^13^C INADEQUATE correlation spectroscopy for the comparison between ^13^C isotopically labeled native cell walls and DNP-assisted ^13^C natural-abundance native cell walls. (**a**) DNP-assisted NMR signals from cellulose and hemicellulose at natural isotope abundance. Superscripts explain the eight types of glucose units in different conformations of xylan. Inset shows the lipid signals, which are folded in the indirect dimension. (**b**) The spectral pattern for ^13^C isotopically labeled native cell walls. (**c**) Arabinose and acetyl signals are shown to be detectable only using ^13^C-labeled samples. (**d**) Comparison between the number of peaks detected using the two different techniques, asterisks indicate the components that are poorly detected using natural-abundance DNP-assisted NMR. Reproduced with permission. Ref. [77] Copyright 2021, Springer.

Another way to visualize cellulose polymorphism in plant cell walls is by using the 2D ^13^C-^13^C INADEQUATE spectrum to collect spectrograms of unlabeled stems of wild-type rice using the DNP system (Figure 3a). At first, this 2D experiment was considered impossible to achieve, because there is a low natural abundance of ^13^C (1.1%) and a lower probability of detecting a cross peak between two carbons. However, now this experiment can be completed using DNP system within 35 h [77]. In cellulose, eight types of glucose units appear: (a) a–e for the glucan chains found in the core of the microfibrils, and (b) f–h for those on the surface. The ^13^C chemical shifts are similar to those found in Arabidopsis, Zea mays, and Brachypodium distachyon [69]. Hemicellulose, on the other hand, shows weak signals. This can be seen in carbon 4 and 5 in threefold xylan (Xn4^3f^ and Xn5^3f^) and carbon 3 and 4 in twofold xylan (Xn3^2f^ and Xn4^2f^). Some weak signals corresponding to α-xylose (x) of xyloglucan can also be seen.

Another advantage of using DNP-enabled 2D spectroscopy is that it has helped resolve many carbon sites in cellulose, polysaccharides, and lipids. The natural abundance of certain carbons is confirmed by DNP, when following the pattern of unlabeled (Figure 3a) and ^13^C-labeled (Figure 3b) rice stems. A few peaks are omitted, including (a) arabinose residues of xylan sidechains; (b) some carbon sites in twofold, threefold, and mixed forms of xylan backbones; and (c) acetyl groups, as shown clearly in Figure 3c. MAS-DNP is efficient for the detection of ordered molecules, such as cellulose carbons and part of xylan backbones, but does not detect arabinose sidechains. This is a result of the wide distribution of conformations of these mobile molecules, resulting in a broadening of their signals [77].

## 3. Lignocellulosic Biomass Structure Interpretation

**Figure 4:**
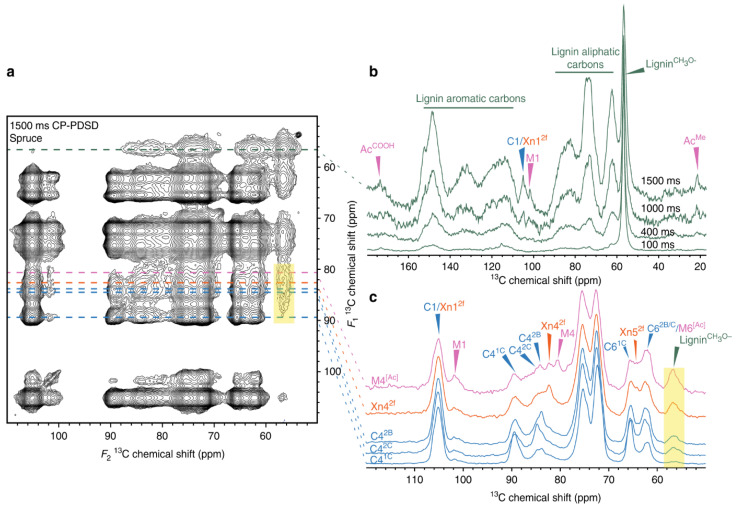
2D ^13^C–^13^C correlation spectroscopy for assisting in locating lignin in spruce. (**a**) The carbohydrate region of a ^13^C CP-PDSD spectrum is shown, with lines marking the 1D slices derived from the 2D spectrum. (**b**) The lignin methoxyl (56.5 ppm) slice extracted at 100, 400, 1000, and 1500 ms mixing times are overlaid and normalized. (**c**) The carbon 4 slices of GGM (80.4 ppm), xylan (82.4 ppm), and three cellulose (2B = 83.7, 2C = 84.5, 1C = 89.5 ppm) sub-domains from the 1500 ms 2D CP-PDSD spectrum are shown, normalized. A translucent yellow box highlights the cross-peak to the lignin methoxyl. Reproduced with permission. Ref. [63] Copyright 2019, Nature Publishing Group.

Lignin is located close to cell wall polysaccharides in structure [93,94]. To confirm this statement, the CP-PDSD spectra for cross-peaks between polysaccharide carbons and lignin methoxyl were examined at 56.5 ppm. In Figure 4a, one can observe the cross peaks of lignin at 56.5 ppm. Figure 4b, slices from varying mixing times at 56.5 ppm slices of spectra are stacked. Up to the 1000 ms mark, the cross-peaks shown are intramolecular from lignin methoxy to aliphatic lignin carbons, which are chemically similar to polysaccharide carbons. Intramolecular cross peaks to aromatic lignin carbons can also be seen at 115–153 ppm. Interestingly, there are no cross-peaks to Ac^Me^ or to polysaccharide carbon 1, which provides evidence that cross peaks between lignin methoxy groups and carbons with chemical shifts 62–90 ppm are intramolecular at the 400 ms mark. At 1000 ms and reaching to 1500 ms, a cross peak between the lignin methoxy group appears, and the galactoglucomannan (GGM) Ac^Me^ and another cross peaks between lignin methoxy group and C1/Xn1^2f^, M1^[Ac]^ appear. This proves that both lignin and the acetylated and unacetylated mannosyl residues in GGM are spatially close. Most of the cross peaks from the lignin methoxy group are intramolecular. This suggests that most of the lignin is distant from polysaccharides [63].

Figure 4c shows slices taken at the carbon 4 shifts for GGM, xylan, and cellulose domains 1C, 2B, and 2C at a 1500 ms mixing time. This was used to determine which of the polysaccharides is closest to lignin. There exists a cross peak between the five carbon 4 nuclei and the lignin methoxy groups at 56.5 ppm; however, there is also a difference between them. To arrange the cross peaks based on strength, we have the strongest detected to M4^[Ac]^ then Xn4^2f^. C4^2b^ and C4^2C^ have a similar intensity, but C4^1C^ shows a very faint signal, being almost undetectable. A general look at these results [94,95] shows that lignin is strongly associated with xylan and GGM bound to cellulose, and some lignin is close to the cellulose surface (cellulose domain 2) [63].

## 4. Effect of Oxidation on Pulp and Viscous Cellulose

The oxidation and functionalization of cellulose has long been investigated [96,97], it has gained much interest in biobased industry, additionally to the formation of several cellulose-based derivatives [98], which were used in different fields, such as drug delivery vehicles [66]. Oxidation involves the selective creation of oxidized groups, mainly as aldehyde functional groups between C_2_ and C_3_ in cellulose, these aldehyde groups serve as anchors for further modification, such as amine molecules (imines) and oxidation to carboxyl groups. Several factors and conditions play an effective role and strongly influence the oxidation rate, such as the oxidative agent type and concentration [99], sonication [100], temperature, ball milling [101], and presence of metal salts [102], in addition to the properties of the native cellulose used, such as the crystallinity, polymorphism, degree of oxidation, and accessibility of oxidizing agent [88].

**Figure 5:**
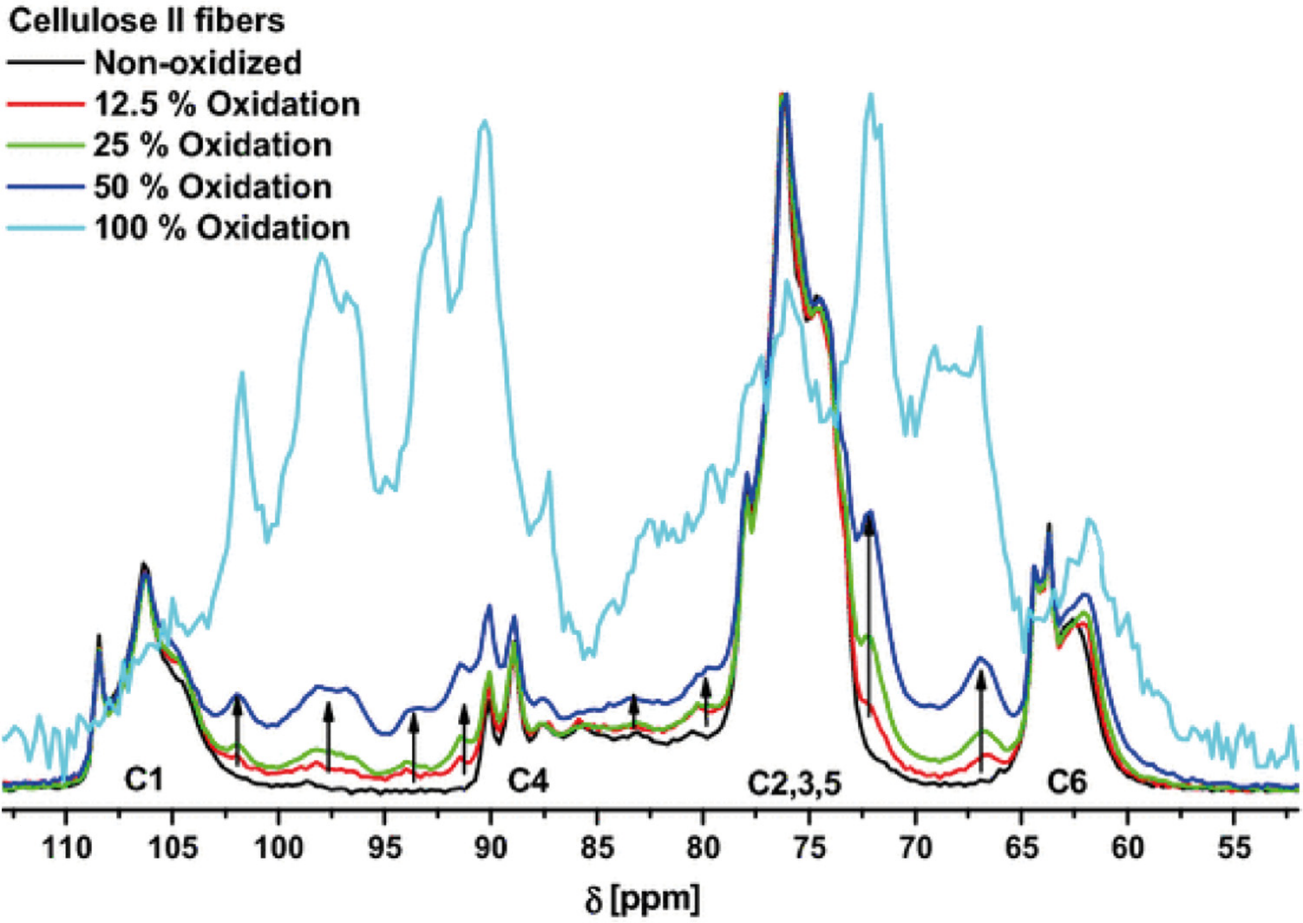
1D ^13^C CP MAS NMR spectra of cellulose II fibers with different periodate oxidation degrees. Reproduced with permission. Ref. [88] Copyright 2015, Springer.

The degree of oxidation for different cellulose polymorphs was studied with ^13^C CP MAS NMR, as shown in Figure 5; the main results include noticeable resonances between 85 and 93 ppm, mainly corresponding to aldehyde hydrates, formation of hemialdal structures between the two newly formed aldehyde groups, having a peak around 97 ppm, which explains the absence of carbonyl resonances between 175 and 185 ppm, and the formation of hemiacetals, showing resonance at 88 ppm, and ones originated from C_6_ between 65 and 69 ppm [88]. Solid state NMR spectroscopy allows a deep insight into the structure of oxidized cellulose, but appears to be less effective and reliable in reporting the crystallinity index [103,104] of the different cellulose polymorphs with the CP MAS technique, due to the overlapping effects appearing on the C_4_ and C_6_ signals and internal crosslinking of hydroxyls from C_6_ [88].

## 5. Production of Crystalline Nanocelluloses via Oxidation of Microcrystalline Cellulose

**Figure 6:**
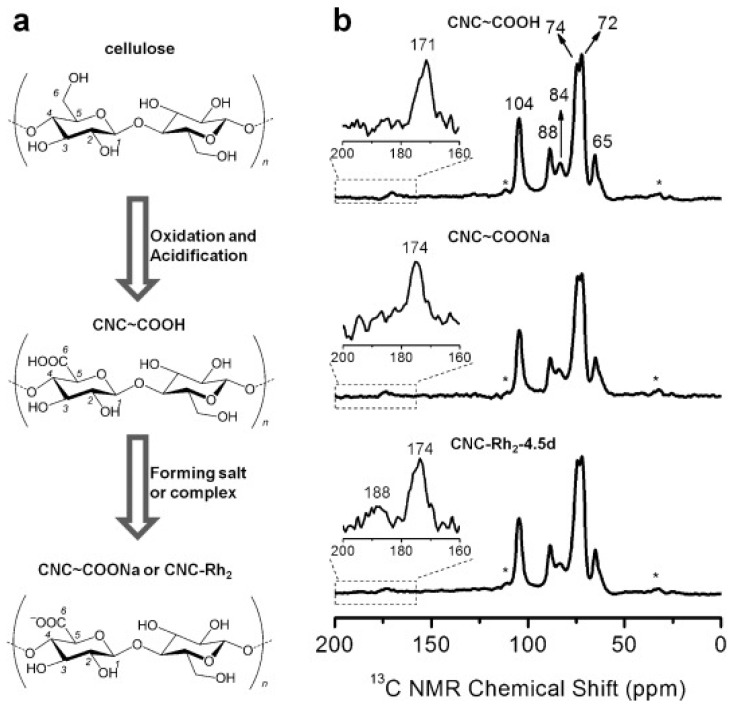
(**a**) Schematic illustration for the CNC synthesis pathway in its COOH and COONa forms. (**b**) 1D ^13^C CP MAS NMR spectra of CNC~COOH (top), CNC~COONa (middle), and CNC-Rh2-4.5d (bottom). Asterisks marked signals indicate spinning side bands. Reproduced with permission. Ref. [111] Copyright 2015, Wiley and Sons.

Microcrystalline cellulose fibers are exposed to TEMPO-mediated oxidation [105], to form a biocompatible and sustainable cellulose-based derivative called crystalline nanocelluloses (Figure 6a), which include the most famous cellulose nanocrystals [106,107] and, due to their biocompatible, sustainable, biodegradable and high surface area properties [105], have attracted researcher attention, to utilize them as reinforcing components [108], drug delivery carriers [66], and supports for nanocatalysts [109]. ^13^C CP MAS NMR spectroscopy [110] is capable of analyzing the chemical environment of cellulose nanocrystals [83] in their different forms of carboxyl groups, in addition to the applied chemical modifications, and comparing these results with the original cellulose [111]. Cellulose nanocrystal spectra (Figure 6b) show carboxyl groups in their acidic form at 171 ppm and sodium binding form at 174 ppm, where the latter also appears in the spectrum of the rhodium modified cellulose nanocrystals, in addition to a newly formed unidentified weak intensity signal at 188 ppm. The newly appearing carboxyl group at 188 ppm confirms that a chemical interaction occurs between the carboxyl group and the surface of the rhodium-based modification [111,112].

## 6. Future Opportunities for Nanocellulose as a Drug Delivery Carrier

**Figure 7:**
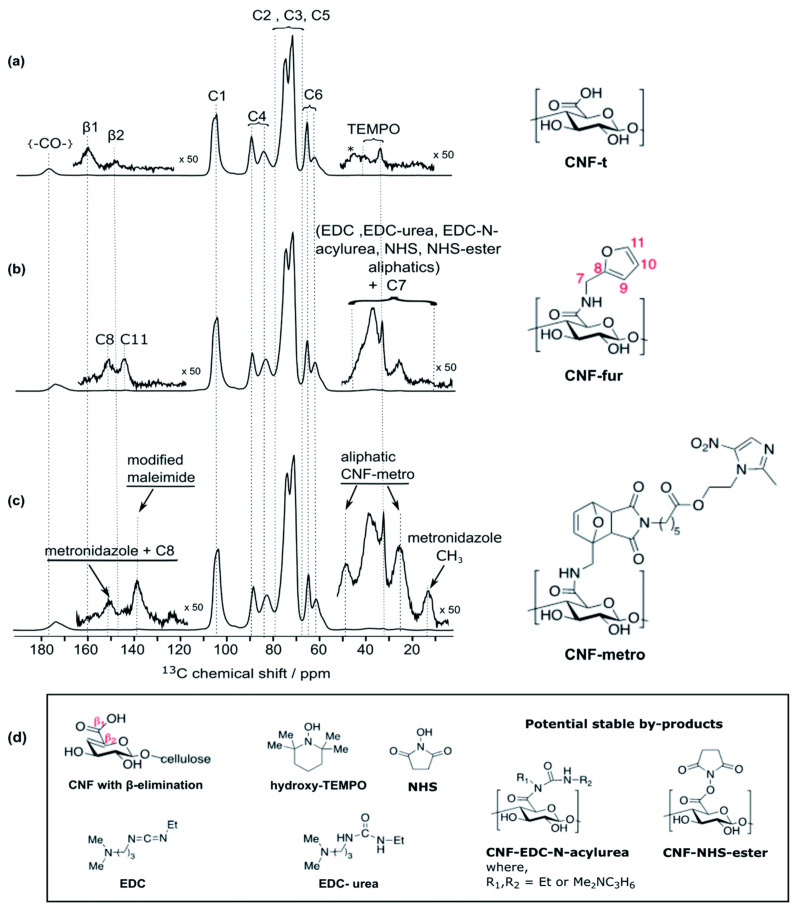
1D ^13^C CP MAS DNP-assisted NMR spectra for surface-modified cellulose nanofibrils, including: (**a**) initial TEMPO-oxidized cellulose nanofibrils (CNF-t), (**b**) furylated cellulose nanofibrils (CNF-fur), and (**c**) maleimide-modified metronidazole on cellulose nanofibrils (CNF-metro). Inserts used in (**a**–**c**) show magnified views of the 0–55 ppm and 115–165 ppm regions. (**d**) Summary for the chemical structures of CNF with b-alkoxy-elimination, hydroxyl TEMPO, the coupling agents EDC and NHS, and their potential stable by-products. Reproduced with permission. Ref. [66] Copyright 2020, The Royal Society of Chemistry.

Nanocellulose has shown promising results and potential applications in the bio-pharmaceutical industry [113], especially due its ability to form different 2D and 3D nano-structures and its stable behavior after exposure to freeze-drying and rehydration [114,115,116]. When drugs are loaded onto cellulose-based nanocarriers, it is then difficult to distinguish between covalently bonded and adsorbed molecules, which leaves open questions in such an intensive field of research. Therefore, DNP-enhanced solid state NMR was used (Figure 7) to study the surface chemistry of nanocellulose drug carriers and overcome the sensitivity and resolution complications in conventional solid state NMR spectroscopy [66]. DNP-enhanced solid state NMR provided additional signals in the aliphatic and aromatic regions over the standard detected signals of nanocellulose fibers by the CP MAS NMR technique, with these newly appearing signals (Figure 7a) corresponding to the methyl groups of reduced TEMPO moieties (10–50 ppm) and carbons (Figure 7b) in the furan ring (C_8_ at 152 ppm, C_11_ at 145 ppm and C9,10 between 105–115 ppm). Moreover, DNP-enhanced solid state NMR was able to detect weak resonance at 148 and 160 ppm, which correspond to cellulose broken chains, since oxidizing the cellulose units occurs under alkaline conditions [66].

The use of DNP-enhanced solid state NMR offered a unique insight into the structure and surface of nanocellulose fibers in their original and drug loaded forms, and was capable of detecting, not only the remaining traces of TEMPO moieties, coupling agents on the surface, and depolymerized cellulosic units, but also precisely estimating the drug loading and distinguishing between adsorption and grafting (covalent bonding). DNP-enhanced solid state NMR proved to be useful, not only for the full characterization of nanocellulose fibers, but also for the understanding of cellulose-based smart drug carriers at natural isotopic abundance [66].

## 7. Summary, Concluding Remarks, and Future Perspectives

**Scheme 2:**
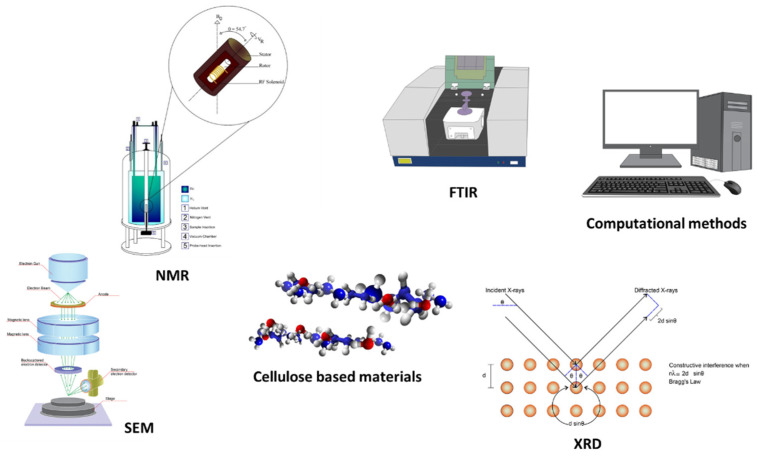
Combined techniques for unravelling the structure of cellulose-based materials.

Solid state NMR has played a key role in different fields of science, including carbohydrate polymers [45,117] and in-particular plant [118] and fungal cell wall [119,120], lignocellulose [93,121] and cellulose-based materials. Solid state NMR spectroscopy, with its modern advancements and pulse sequences, is a promising technique for resolving as yet missing aspects of the molecular structure and conformation, polymorphism, wettability, water-matrix interactions, packing, and dynamics of cellulose-based materials. In this article, we have reviewed the most advanced NMR strategies and pulse sequences used in studying cellulose-based materials and highlighted the benefits gained from the usage of every set of pulse sequences. Additionally, advanced in-situ NMR experiments can be highlighted as promising for cellulose-based materials, such as variable temperature T_1_, T_2_ relaxation and linewidth analysis used for studying polymorphism, polymeric chain dynamics, and water–matrix interactions; advanced water-edited 1D ^13^C and 2D ^13^C-^13^C CP MAS based experiments used to detect the proximity and accessibility of water to the different cell wall components; pulse field gradient NMR experiments used for investigating the dissolution process and ion dynamics in different cellulose structure; and the comprehensive multiphase NMR technique, which is basically a combination of solid and solution state NMR techniques, and used to follow the processes involving the conversion of solid to liquids and vice versa, including drying, swelling, conformational changes, and dissolution processes in cellulose-based materials. Many issues and challenges in solid state NMR spectroscopy still undervalue the importance of such an instrument, such as its resolution and sensitivity, but recent technical and hardware advancements have provided exceptional solutions. Nowadays, solid state NMR advanced technologies and high-tech opportunities beyond the conventional methods, including advanced hyperpolarization techniques (especially Dynamic nuclear polarization); sensitivity boosting techniques via isotopic enrichment; paramagnetic doping and the usage of CryoProbes; magnetic resonance imaging and microimaging; NMR relaxometry; NMR diffusometry; ultra-high magnetic fields; and ultra-fast MAS spinning are able to provide deep insights, not only into the primary structure of cellulose, but also into the reaction mechanisms occurring in the structure, such as detecting intermediates appearing during oxidation and functionalization of cellulose, tracing the drug delivery release processes, and monitoring the water distribution, transport, and dynamics. There is no single analytical technique capable of providing information on all different chemical levels. Ideally, the pursuit of integrated methods, such as the combination of solid state NMR advanced techniques and computational approaches [122,123], can provide the most valuable information and deep insights in studying cellulose-based materials. Hence, the optimum goal is to combine various characterization methods coupled to NMR to unravel the structure of cellulose based materials, as depicted in Scheme 2.

## Figures and Tables

**Table 1:** A summary for the most important pulse sequences and techniques used in the structural and dynamical analysis of cellulose-based materials.

Pulse Sequence	Applications	References
1D ^1^H Static NMR	Detection of cellulose I different phases	[56]
1D ^1^H ^a^ CRAMPS	High-resolution spectra for rigid solids and inter-molecular interactions	[57]
1D ^1^H Depth	Indicating rigid structures (chain dynamics)	[57]
1D ^31^P MAS	Structural confirmation of cellulose phosphorylation	[58]
1D ^13^C CP MAS	Structure analysis of cellulose, quantification of cellulose in blends, determination of degree of substitution	[59,60,61]
1D ^13^C CP MAS, ^b^ DANTE selective excitation pulse trains	Selective excitation of the C4 as a function of the diffusion time	[62]
1D ^13^C CP-^c^ PSRE MAS	Detecting cellulose crystallite thickness	[38]
1D ^13^C CP-^d^ PDSD MAS	Cellulose structure analysis and cross-peak determination with lignin	[63]
1D ^13^C MultiCP MAS	Quantitative cellulose crystallinity	[64]
1D ^13^C MultiCP MAS with dipolar filter	Filtering spectra for hemicellulose and lignin (spectral editing)	[64]
Paramagnetic relaxation enhancement 1D ^13^C T_1_ CP MAS	Solvent accessibility of different fractions of wetted cellulose	[65]
^g^ DNP enhanced 1D ^13^C CP MAS	Surface chemistry of cellulose and nanocellulose	[66,67]
Polarization transfer solid state NMR (^e^ DP, CP and ^f^ INEPT)	Studying of cellulose dissolution	[68]
Polarization transfer solid state NMR (T1, T2 filter)	Water proximity to cellulose, hemicellulose and pectin. Water mobility	[69,70]
Inversion recovery CP excitation and Saturation recovery	Estimation of ^13^C T_1_ relaxation times and crystallinity of cellulose	[71]
Relaxation measurements by Torchia-CP	Modified method for measuring ^13^C T_1_ relaxation times	[72]
Rotor synchronized MAS	Investigating the molecular orientation distribution	[73]
^7^Li and ^23^Na ^h^ PFG-SE NMR	Studying of cellulose dissolution, provide proximity information between cations/macromolecule	[74]
2D ^13^C-^13^C ^i^ INADEQUATE by ^g^ DNP	Atomic resolution structural analysis without isotopic labeling	[75,76,77]
2D ^13^C-^13^C ^i^ INADEQUATE by ^13^C isotopic labeling	Structural connectivity determination	[78,79]
2D ^13^C-^13^C ^d^ PDSD/^j^ DARR	Structural connectivity determination	[51,78]
2D ^1^H-^6^Li,^13^C ^k^ LGHETCOR	Studying of cellulose dissolution, provide proximity information between covalent bonded ^1^H-^13^C spins, and probing remote ^1^H-^13^C correlations	[80]
2D ^13^C ^d^ PDSD experiments	Multi-bond and long range inter-molecular cross peaks	[69,70]
2D CHHC correlation experiments	Determination of the hydroxylmethyl conformations	[81]
2D ^l^ RFDR correlation experiments	Assigning the intra-residue cross peaks	[69]
2D 1H-^13^C ^m^ WISE	Indicating molecular mobility and water localization in blends	[57]
^13^C-^15^N ^n^ REDOR experiments	Indicate distance between carboxyl carbon and nitrogen of the modification	[82]
^2^H-^13^C ^n^ REDOR experiments	Detection of the ^1^H-^2^H exchangeable regions in cellulose	[83]
^o^ CPMG Time domain NMR	Identifying different water phases in hydrolyzed cellulose	[84]
^o^ CPMG Cryoporometry NMR	Determination of pore volume, radius and size distribution	[85]

^a^ CRAMPS (combined rotation and multiple pulse spectroscopy); ^b^ DANTE (delay alternating with nutation for tailored excitation); ^c^ PSRE (proton spin relaxation edition); ^d^ PDSD (proton driven spin diffusion); ^e^ DP (direct polarization); ^f^ INEPT (insensitive nuclei enhanced by polarization transfer); ^g^ DNP (dynamic nuclear polarization); ^h^ PFG SE (pulse field gradient spin echo); ^i^ INADEQUATE (Incredible Natural Abundance Double Quantum Transfer Experiment); ^j^ DARR (dipolar assisted rotational); ^k^ LGHETCOR (Lee Goldberg heteronuclear correlation) ; ^l^ RFDR (radio frequency driven recoupling); ^m^ WISE (wide line separation experiments); ^n^ REDOR (rotational echo double resonance); ^o^ CPMG (Carr Purcell Meiboom).
